# Demoralization, Patient Activation, and the Outcome of Spine Surgery

**DOI:** 10.3390/healthcare4010011

**Published:** 2016-01-19

**Authors:** Andrew R Block

**Affiliations:** Texas Back Institute, Plano, TX 75093, USA; ablock@texasback.com; Tel.: +1-972-608-5000

**Keywords:** presurgical psychological screening, spine surgery, demoralization, patient activation, MMPI-2-RF, maximizing factors

## Abstract

It is now well established that psychosocial factors can adversely impact the outcome of spine surgery. This article discusses in detail one such recently-identified “risk” factor: demoralization. Several studies conducted by the author indicate that demoralization, an emotional construct distinct from depression, is associated with poorer pain reduction, less functional improvement and decreased satisfaction among spine surgery patients. However, there are indications that the adverse impact of risk factors such as demoralization can be mitigated by psychosocial “maximizing” factors—characteristics that propel the patient towards positive surgical results. One of these maximizing factors, patient activation, is discussed in depth. The patient activation measure (PAM), an inventory assessing the extent to which patients are active and engaged in their health care, is associated not only with improved spine surgery results, but with better outcomes across a broad range of medical conditions. Other maximizing factors are discussed in this article. The author concludes that the past research focus on psychosocial risk factors has limited the value of presurgical psychological screening, and that future research, as well as clinical assessment, should recognize that the importance of evaluating patients’ strengths as well as their vulnerabilities.

## 1. Introduction

The use of surgery for protracted back and neck pain has increased rapidly over the past two decades, with spinal fusion accounting for a majority of this growth. For example, Rajaee *et al.* [1] examining the United States data set for the Healthcare Cost and Utilization Project (HCUP) for the years 1998 to 2008, found that spinal fusion increased 2.4 fold from 174,223 to 413,171. The average hospital costs associated with such surgeries have also increased significantly, as reported by Weinstein *et al.* [2] who found the hospital costs associated with spinal fusion averaged about $82,000 per patient, a 3.3 fold increase compared to 1998. By 2008 national hospital billing for spinal fusion alone was almost $34 billion.

Spine surgery can be, and often is, quite effective. For example, Weinstein *et al.* [3] in the Spine Patient Outcomes Research Trial (SPORT) study found that at both six months and two years post-op, patients undergoing laminectomy/discectomy obtained greater reductions in pain and improvements in functional ability than did patients treated non-surgically for herniated lumbar discs. Similarly, Mirza *et al.* [4] found that individual with back pain who underwent spine surgery (mostly spinal fusion) showed greater reduction in disability than did those who did not receive invasive treatment (see also Fritzell *et al*. [5]).

On the other hand, it is well established that spine surgery not infrequently fails to provide pain relief and improved functional ability. For example, a recent analysis of discectomy patients found 28% had unfavorable outcomes [6], with a 10% reoperation rate. In like fashion, Copay *et al.* [7] found that of patients who had undergone spine surgery (mostly spinal fusions) only 47% to 61% showed clinically significant improvement on any one of the outcome measures examined. Further, Brox *et al.* [8] found that long-term improvement after instrumented fusion was no better than treament with a combination of cognitive-behavioral intervention and exercise (see Mirza and Deyo [9] for a systematic review).

A large and growing body of research is finding that psychosocial risk factors can contribute significantly to the variability in spine surgery outcome (for reviews see Block [10]; Block, Gatchel, Deardorff and Guyer [11]. Research by our group (Block *et al*. [12]; Marek, Block and Ben-Porath [13]), and others (e.g., Edwards *et al*. [14]; Trief, Grant and Fredrickson [15]; Voohies, Jaing and Thomas [16]; Chiachana *et al.* [17]) demonstrate the adverse impact on surgery results of depression, elevated pain sensitivity, workers’ compensation, somatic anxiety and poor pain coping, to name just a few. In one of our studies (Block *et al*. [18]) patients identified as having a high level of psychosocial risk had only about a 15% chance of obtaining good surgical outcome, as defined by reduction in pain and improvement in functional ability.

## 2. Demoralization

Until recently, much of the research on presurgical psychological screening (PPS), was based on the use of the original Minnesota Multiphasic Personality Inventory (Hathaway and McKinley [19]) or its first major revision, the MMPI-2. However, recent research by our group using the latest revision of this test, the MMPI-2-Restructured Form (MMPI-2-RF: Ben-Porath and Tellegan [20]) has identified another psychological factor, demoralization, that is powerfully associated with reduced outcome of both spine surgery (Marek, Block and Ben-Porath [13]) and spinal cord stimulation (Block, Marek and Ben-Porath [12]). Ben-Porath [21] defines demoralization as “a pervasive and affect-laden dimension of unhappiness and dissatisfaction with life”. Demoralization, assessed by a 24-item scale exclusive to the MMPI-2-RF, scale RCd, includes items that “reflect the presence of dysphoric affect, distress, self-attributed inefficacy, low self-esteem and a sense of giving up” (p. 53). Such feelings appear to underlie a broad range of mental health disorders. For example, Simms *et al.* [22] found that among military veterans RCd elevations correlate strongly with both current and lifetime diagnosis of depressive and anxiety disorders, and with negative emotionality. Scale RCd, demonstrates desirable psychometric properties, including strong test-retest reliability r^2^ = 0.88, and internal consistency (r^2^ ranging from 0.87 to 0.93 depending on the population tested), with no significant differences between average scores of men and women (Tellegen and Ben-Porath [23], pp. 24–25).

Our research (Marek *et al*. [13]; Block, Marek, Ben-Porath and Ohnmeiss [24]) has found that elevated scores on the demoralization scale, RCd, are strongly correlated with poorer results at six months post spine surgery, including less improvement in pain and in self-reported physical disability, lower return to work rates, greater use of opioid medication, poorer satisfaction with surgical outcome, and worse overall outcome. Further, specific components of demoralization assessed by the MMPI-2-RF, including scales measuring Helplessness/Hopelessness, Self-Doubt and Inefficacy (a belief that one is incapable of making decisions and coping with difficulties), are significantly associated with poorer satisfaction and reduced results of both spine surgery (Block, Ben-Porath, Marek and Ohnmeiss [24]) and poorer results of spinal cord stimulation (Block, Marek, Ben-Porath and Kukal [12]). Further, scale RCd, is the only MMPI-2-RF scale associated with poor results in all the outcome areas assessed. For spinal cord stimulator candidates [12] T-scores of 60 (1 standard deviation above the mean) or greater on scale RCd significantly increased the relative risk ratio (RRR) for poor results on all measures utilized, including functional ability as assessed by the Oswestry Disability Index (RRR = 1.42), reported pain level (RRR = 1.47) and patient rating of dissatisfaction with outcome (RRR = 1.86). Unpublished data [25] by our group indicate similarly increased RRRs for poor outcome in spine surgery candidates.

Elevated scores on scale RCd have also been found to be associated with poorer conservative treatment outcomes in chronic low back pain. Tarescavage, Scheman and Ben-Porath [26] examining the effectiveness of an interdisciplinary treatment program for chronic low back pain, found significant correlations between scores on scale RCd with emotional distress and pain-related disability at the completion of the program.

A substantial literature exists on demoralization, especially in the context of other chronic medical conditions. Most of these studies use instruments other than the MMPI-2-RF, including the Diagnostic Criteria for Psychosomatic Research (DCPR: Fava *et al.* [27]) or the Demoralization Scale (DS: Kissane *et al.* [28]). A recent systematic review (Robinson *et al.* [29]) found that for individuals with a wide range of chronic illness and disease 13%–33% experience demoralization (depending on the measure utilized to assess this condition), and that demoralization is associated with poorly controlled physical symptoms, including fatigue, mobility constraints, breathing problems, constipation, memory and concentration problems. Further, for the medically ill patients studied there was also a strong negative association between activity level and demoralization.

Demoralization is distinct from depression, although both may include strong experience of negative emotions. Individuals who are depressed, in addition to displaying vegetative symptoms such as sleep disturbance, psychomotor retardation and lethargy, exhibit anhedonia, *i.e.*, inability to experience pleasure (de Figueiredo [30]). Demoralized individuals, on the other hand, can experience positive emotion, but are plagued by feelings of helplessness, loss of hope and meaninglessness (Sansone and Sansone [31]). Several studies have documented the divergence of demoralization and depression. Grandi, Sirri, Tossani and Fava [32] examining cardiac transplant patients, found that 71% of patients who were determined to be demoralized according to the DCPR did not fit the criteria for major depression. Similarly, Jacobsen *et al.* [33] found among patients with advanced cancer that, of those diagnosed with major depressive disorder, only 28.6% met the DCPR criteria for demoralization. While it is clear that spine surgery results are diminished in patients reporting high levels of depression (Chaichana *et al.* [17]; Adogwa *et al.* [34]), which is often assessed using the Zung depression inventory (Zung [35]), demoralization is a distinctive emotional state, and one which appears to exert particularly adverse effects on medical conditions in general, and spine surgery in particular.

## 3. Patient Activation

The feelings of ineffectiveness, helplessness and the sense of giving up that comprise the core of demoralization stand in sharp contrast to the behaviors and general health orientation that are associated with positive health outcomes. In order to achieve and maintain good health, individuals must be able take control over diet and exercise and seek out health information. Individuals also need to recognize when illness occurs, and be able to communicate with health care providers. They need to work with their physicians on plans to overcome or mitigate illness, and have the fortitude to follow through on these plans. Such an effective health orientation is captured by the Patient Activation Measure (PAM: Hibbard, Stockard, Mahoney and Tissler [36]).

The PAM is a 20-item questionnaire designed to assess the extent to which individuals are “engaged and active” in their own health care. The domains evaluated by the PAM include: (1). Belief that taking an active role in health is important; (2). Having the confidence and knowledge to take action; (3). Taking health-related action; (4). Staying the course under stress. In the original studies, PAM scores correlated significantly with the use of a glucose journal in diabetes, with following a low fat diet in patients with high cholesterol, with routinely exercising for patients with arthritis, and for seeking out information from health care providers. Further studies have found the PAM correlates significantly with both health outcomes and health care utilization. For example, in diabetics PAM scores predicted testing for, and control of Hemoglobin A_1c_, and testing for low-density lipoprotein cholesterol, among others (Remmers, Hibbard, Mosen, Wagenfield, Hoye and Jones [37]). In an analysis of over 33,000 patients in a large health care delivery system in Minnesota, patients with the lowest scores on PAM (poorest patient activation as determined by their scores being in the lowest quartile) had much higher average health care costs than patients who displayed the highest levels of patient activation, a finding which held true not only for population as a whole, but for specific groups of patients, including those with hyperlipidemia, hypertension, and asthma (Hibbard, Green and Overton [38]).

Two previous studies have examined the role of patient activation in spine surgery patients. Skolasky, Mackenzie, Wegener and Riley [39] examined 65 patients who underwent surgery for degenerative lumbar stenosis, assessing the relationship of PAM scores to participation in post-operative physical therapy (PT). They found that PAM scores correlated strongly with both the number of PT sessions attended, and with patient “engagement” in PT, as assessed by the physical therapist using a standardized treatment engagement metric. Skolasky, Mackenzie, Wegener and Riley [40] went on to examine the relationship of patient activation to functional recovery in spine surgery patients. In this study, patients in the highest level (upper quartile) of PAM scores showed greater reduction in reported pain levels at post-op follow up than did patients with lower levels of patient activation, despite the fact that the patients with highest PAM levels reported less pain at baseline. Patients in the upper quartile of PAM scores also showed the greatest improvement in functional ability, as assessed by the Oswestry disability index (Fairbank [41]), and the greatest improvements in overall physical health as assessed by the SF-12 v 2 (Hurst, Duta and Kind [42]). The authors conclude that including interventions to improve patient activation, such as empowerment strategies, self-management strategies and education sessions, may lead to improvements in the outcome of spine surgery.

We have been examining the relationship of the PAM to the outcome of spine surgery. Thus far, we (Block, *et al*. Unpublished data [25]) have given the PAM, as well as the MMPI-2-RF to a group of patients prior to surgery (both spine surgery and spinal cord stimulator implantation), finding significant correlations of the PAM with improvements in functional ability as assessed by changes in scores on the Oswestry disability index (r^2^ = 0.33, *p* < 0.01 ), reduction of negative affect as assessed by change scores on Likert-type emotion ratings (r^2^ = 0.26, *p* < 0.05), and with patient satisfaction (r^2^ = 0.28, *p* < 0.05) at an average of about 5 months post-op.

## 4. Psychological “Maximizing” Factors

Results with the PAM point to a very significant and long-neglected area in presurgical psychological screening of spine surgery candidates, *viz*, the assessment of patient characteristics that may militate towards improved outcomes. Certainly, my own research (see Block [10]) as well as that of others (Voorhies *et al.* [16]; denBoer *et al.* [43]), which has been focused on assessment of psychological “risk” factors, continues to demonstrate how specific psychological characteristics can undo even the most effective surgical intervention. We (Block, Marek, Ben-Porath and Ohnmeiss [24]) have found that, in addition to demoralization, several other characteristics assessed by the MMPI-2-RF are strongly correlated with reduced spine surgery results, including somatic sensitivity and malaise (Scales RC1 and MLS), low positive emotion (scale RC7), family problems (FML), social avoidance (SAV), and the PSY-5 scale negative emotionality/neuroticism (NEGE-r). However, the complexity of human nature is such that individuals may have strengths—traits, behaviors and emotional states--that can counteract more negative characteristics. Patient activation may be one such “maximizing factor”—one that could potentially reduce the adverse impact on some psychosocial risk factors, such as demoralization, on spine surgery results.

Other potential “maximizing factors” warrant exploration. Consider, for example, a patient who has a high level of family problems (elevated score on the MMPI-2-RF scale FML). Such a patient may simultaneously have a strong social support system outside the family, or even be satisfied with the level of support received by family members, despite the problems that exist within the family. Social support has been found to be an important predictor of improved health outcomes. For example, Mutran, Reitez, Mossey and Fernandez [44], examining recovery from hip surgery, found that patients with low levels of perceived support achieved less improvement in walking ability at 2 months post-op than did patients with higher level of support. In the case of spine surgery, Schade *et al*. [45] found that social support from the spouse was significantly related to greater pain relief in patients undergoing lumbar discectomy. Further, higher levels of perceived support have been found to be associated with less catastrophizing in patients who have longer pain durations (Cano [46]). Thus, it appears that the perception of satisfactory social support may be a factor that is associated with improved surgical outcome, and one which may mitigate some psychosocial risk factors, including catastrophizing and elevated levels of family problems.

A third potential characteristic that may help to maximize spine surgery results revolves around expectations for the outcome of spine surgery. Spine surgery has three major goals: reduce pain; improve functional ability; and correct the underlying physical pathology responsible for the pain and functional deficits. The extent to which these three goals are achieved, however, varies widely. Some patients coming to surgery expect total pain relief and a complete return to pre-morbid activity levels, while others may consent to surgery even though their expectation is that minimal improvements will occur. So, it is reasonable to consider whether patient expectations bear a relationship to surgical results. Although the results are not completely consistent, several studies show that greater expectations of improvement assessed pre-operatively are associated with more sanguine surgical results. For example, Yee *et al*. [47] examining spinal fusion patients, found that higher preoperative expectations were associated with greater improvement on the SF-36 physical domain score. Similarly, Soroceanu, Ching, Abdu and McGuire [48]) found higher outcome expectations to be associated with greater functional improvement (but not greater satisfaction) in a mixed group of patients undergoing lumbar and cervical spine surgery. Gepstein *et al.* [49] examining elderly patients who went decompression surgery for lumbar spinal stenosis found that positive outcome expectations were associated with greater outcome satisfaction. However, it is clear that having excessively optimistic expectations may work against surgical results and satisfaction. Patients whose high surgical expectations are not met report very low satisfaction with surgery outcome (Toyone *et al*. [50]). Thus, it appears that surgical success is more likely to be achieved when patients have expectations of significant, but not complete, pain relief and substantial, but not completely unrestricted, improvement in functional ability.

Patient activation, social support and positive outcome expectations are but three of a host of psychosocial factors that might potentially be associated with improved spine surgery results. Some other factors that have been found to correlate with improved outcome of treatment for pain, and may militate towards better spine surgery response include:

-- Positive pain coping strategies, such as optimism (Goodin and Bulls [51]; Bargiel-Matusicwicz and Kryzyskowska [52]), acceptance and mindfulness (McCracken and Vowels [53]);
-- Resilience (Sturgeon and Zaruta [54]; Ramirez-Maestre, *et al.* [55]) and Hardiness (Maddi [56]);
-- Spirituality and forgiveness (Rippentropp *et al.* [57]).

It would be of great value to explore these and other positive factors that may contribute to better spine surgery results.

## 5. Conclusions

A number of psychosocial risk factors for reduced spine surgery outcome are by now well established. Depression, somatic sensitivity, demoralization, substance abuse, vocational issues such as workers’ compensation and litigation—all these are shown to have strong empirically-derived correlations with diminished results. However, the focus of PPS upon psychosocial risk factors has ignored much of the complexity of each case and provided limited insight into factors that may improve surgical outcomes. Research on patient activation, social support and surgical outcome expectations point to the importance of examining psychosocial “maximizing factors”—those patient characteristics that may mitigate the adverse impact of established risk factors, and may propel the patient towards good surgical response. In order to provide a full and effective picture of each patient’s capacity for achieving reduction in pain and improvement in functional ability, the field of presurgical psychological screening must begin to focus as much on the patient’s strengths as upon his or her vulnerabilities.
